# Maternal mortality in Spain and its association with country of origin: cross-sectional study during the period 1999–2015

**DOI:** 10.1186/s12889-018-6091-4

**Published:** 2018-10-11

**Authors:** V Blagoeva Atanasova, J Arevalo-Serrano, E Antolin Alvarado, Santiago García-Tizón Larroca

**Affiliations:** 1 Department of Obstetrics and Gynaecology, Hospital General Universitario Gregorio Marañón, Universidad Complutense de Madrid, Madrid, Spain; 2 Department of Internal Medicine, Hospital Universitario, Principe de Asturias de Alcalá de Henares, Madrid, Spain; 3 Department of Obstetrics, La Paz University Hospital, Madrid, Spain; 4 Obstetrics and Gynecology Unit, Department of Obstetrics and Gynaecology, Hospital General Universitario Gregorio Marañón, Universidad Complutense de Madrid, 28029 Madrid, ES Spain

**Keywords:** Maternal mortality, Ethnic groups, Spain, Epidemiology

## Abstract

**Background:**

The available literature suggests that there are significant differences in maternal mortality according to maternal origin in high income countries. The objective of this study was to quantify the risk of maternal death by maternal origin and region of Spain where the birth occurred and to identify the most important causes of maternal death in our country.

**Methods:**

An ecological cross-sectional study was conducted that included all deliveries that resulted in maternal survival and cases of maternal death during 1999–2015 in Spain. A descriptive analysis of the maternal mortality rate by maternal origin, region and year of birth was performed. The risk of maternal death was calculated using univariate and multivariate logistic regression analysis, with adjustment for the variables included in the descriptive analysis.

**Results:**

There were 272 maternal deaths during this period, most of which were due to haemorrhage (63 cases, 23.16%).Women whose continent of origin was South America had the highest adjusted risk of maternal death, with an OR of 3.92 (95% CI 2.75–5.58). The region of Spain with the highest risk of maternal death was Ceuta, with an OR of 12.11 (95% CI 2.02–72.68).

**Conclusions:**

This study shows that there are inequalities in maternal mortality according to maternal origin and region where labour occurred. These findings highlight the need to establish strategies at the national and European levels to analyse the most relevant causes and risk factors associated with maternal mortality in order to reduce it and pay closer attention in identifying and carefully managing pregnant women from this at risk groups.

## Background

Maternal mortality remains unacceptably high worldwide, with an estimation of 303,000 deaths each year as a result of pregnancy and birth complications.

There is a substantial lack of progress in the reduction of this adverse outcome due to complex and various factors. In 2015, the World Health Organization (WHO) released “Strategies toward ending preventable maternal mortality (EPMM)” (EPMM Strategies), a direction-setting report indicating global targets and strategies for reducing maternal mortality in the Sustainable Development Goal (SDG) period [1].

Say L et al. claimed that many maternal deaths do not have well identified causes. Using the available data between 2003 and 2009 almost 73% of all maternal deaths were due to direct obstetric causes [2].

The number of births in Spain has decreased progressively since 1940, although the patterns have differed across years and regions. Currently, the ability of families to limit the number of children and to choose the right time to have them through the use of contraceptive methods defines the birth rate in our country. Thus, we can say that Spain has one of the lowest birth rates in the world; this can be attributed to the economic crisis that occurred in the 1970s, as well as to changes in the customs and habits of Spanish couples that persisted despite improvements in the country’s economy in later decades [3].

There are multiple social, economic and cultural factors related to women’s lifestyle that influence their reproductive capacity, the monitoring of their pregnancy and the obstetric outcome of their pregnancies in European Union countries such as ours. Immigration is one such factor.

The impact on health care of migrants displaced to European countries due to economic instability or humanitarian crises in places where there are armed conflicts has been studied before [4, 5]. Some large cities, such as Barcelona or Madrid, have seen their population grow in certain neighbourhoods, where immigrants account for up to 44% of residents. Immigration has also led to an increase in the annual birth rate by almost 20% over 15 years in some of these regions [6].

Risk profiles among immigrant women differ according to the political, social and economic characteristics of the country of origin. Some authors have proposed using sociological indexes such as the HDI (Human Development Index) to define the risks of adverse perinatal events for each pregnant woman depending on the country of origin [7, 8]. Many research studies in obstetrics have focused on immigrant women. Several of these studies have shown an increased risk of adverse perinatal outcomes [9], whereas others have shown a protective effect [10, 11].

This heterogeneity in the outcomes of pregnancies among immigrants when compared with native women may be due to the use of different methodologies in each study as well as to inadequate evaluation of variables that define the socioeconomic situation of patients, such as race or non-native origin, regardless of educational level or degree of deprivation [12].

The economic crisis that began in 2008 in Spain has also led to greater economic inequality among its citizens [13]. This crisis has led to legislative changes that restricted universal access to health care for illegal immigrants [14]. The impact of these measures on the health of pregnant women who come from more impoverished countries remains unclear. Although it is possible that in rich countries, such as ours, improvements in health care can adequately withstand cycles of greater economic recession, this has not been studied in our country.

One of the Millennium Development Goals set in the year 2000 by the member countries of the United Nations is to improve the health of women through multiple interventions such as promoting access to family planning services and emergency obstetric care in the hands of qualified and trained personnel. In this respect, women from low-income countries are especially vulnerable to death due to obstetric causes in their places of origin [15]. However, in several high income countries, such as the United States, there are significant differences in maternal mortality, with greater risk among non-Hispanic black women compared to white pregnant women [16–18].

Several possible causes of this disparity in maternal mortality according to ethnic origin have been proposed, including the higher rate of obesity and cardiovascular risk factors in the immigrant population [19] or differences in prenatal care and adherence to pregnancy monitoring programmes [20].

Recent studies claimed that the risk of maternal mortality and severe morbidity among migrants in high income countries is increased compared to host population. Moreover, an emerging popularity of anti-immigrant political measures in several European countries may worsen migrants vulnerability, especially in pregnants [21].

It is not clear whether immigrant women have a higher risk of maternal mortality in Spain, and there is a need to quantify this risk according to their origin and the region of Spain where the birth occurred. An additional objective of our research is to identify the most important causes of maternal death in our country.

## Methods

This is a cross-sectional study that includes all live births and cases of maternal death during the period of 1999–2015 in Spain. Data were provided by the Spanish National Institute of Statistics (Instituto Nacional de Estadistica - INE), which specified the country of maternal origin of each woman who gave birth and the region and city where the birth occurred. Every maternal death must be reported to the INE through three documents in our country; the death medical certificate which is filled out by the healthcare professional with the cause and the date of the maternal death according to the latest WHO recommendations, the statistical bulletin of judicial death and the statistical bulletin of childbirth.

In the sample of cases that resulted in maternal death, information was also provided regarding maternal age and the cause of death coded according to the ICD-10 classification. The study variables were therefore the cause of maternal death, the country of maternal origin, the year in which the birth occurred and the city and the region where the birth occurred.

The patients were subclassified into 7 groups according to continent of maternal origin:

- Asia
- Western Europe
- Eastern Europe
- North America
- South America
- Africa
- Oceania

The definition of maternal death used in this study was that given by the World Health Organization (WHO) [22]: “The death of a woman while she is pregnant or within 42 days after the termination of the pregnancy, regardless of the duration and site of pregnancy, due to any cause related to or aggravated by the pregnancy itself or its care, but not due to accidental or incidental causes”.

### Statistical analysis

First, a descriptive analysis of maternal mortality was carried out. Variables included continent of maternal origin, region where the birth occurred and cause of death according to ICD-10 classification. The maternal mortality ratio was calculated as the rate between the number of maternal deaths observed during this period of time in Spain and the total number of live births, expressed per 100,000 live births; this represents the risk of maternal death with respect to the number of live births.

We performed a logistic regression analysis using the following categorical variables: region of Spain where the birth occurred, continent of maternal origin and year in which the birth occurred. Indicator variables were used, with the category with the lowest maternal mortality serving as the reference.

Univariate analyses with each of the 3 predictor variables and a multivariate analysis with all 3 variables were performed. The results were expressed as odds ratios (ORs) with a 95% confidence interval, and *p* values < 0.05 were considered significant.

All statistical analyses were carried out using STATA version 15.0 (Stata Corp, College Station, TX). Part of the methodology of this study is based on the previous study by Luque Fernandez MA et al. [23].

## Results

**Fig. 1:**
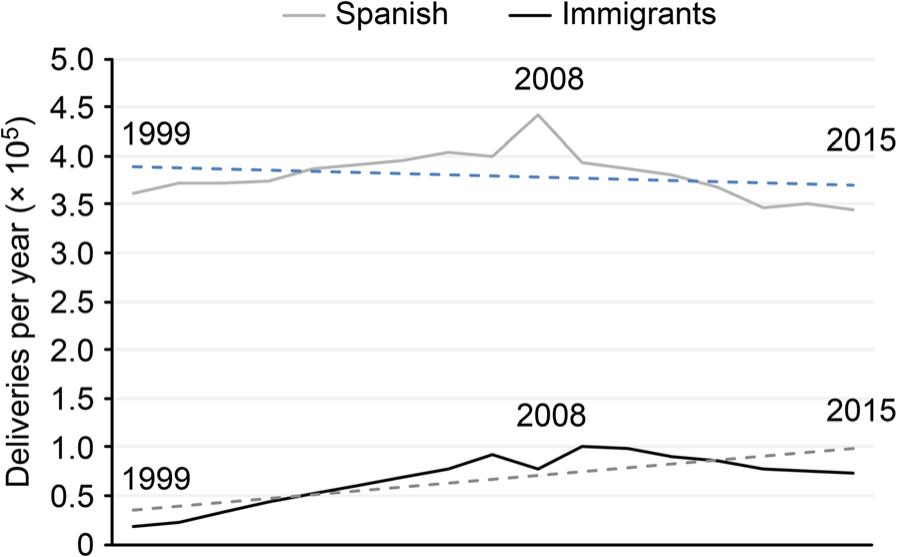
Evolution of live births in Spain 1999–2015

There has been a slight increase in the annual number of births in Spain, with a peak in 2008 (518,188 births) and an average of 447,934 ± 39,019 births/year during the study period. This increase is due to the greater number of births occurring among immigrant women (Fig. 1). There were 17,577 (4.6%) deliveries among mothers of foreign origin in 1999, compared to 73,428 (17.5%) in 2015, with a similar trend in the last 5 years of the study period.

**Fig. 2:**
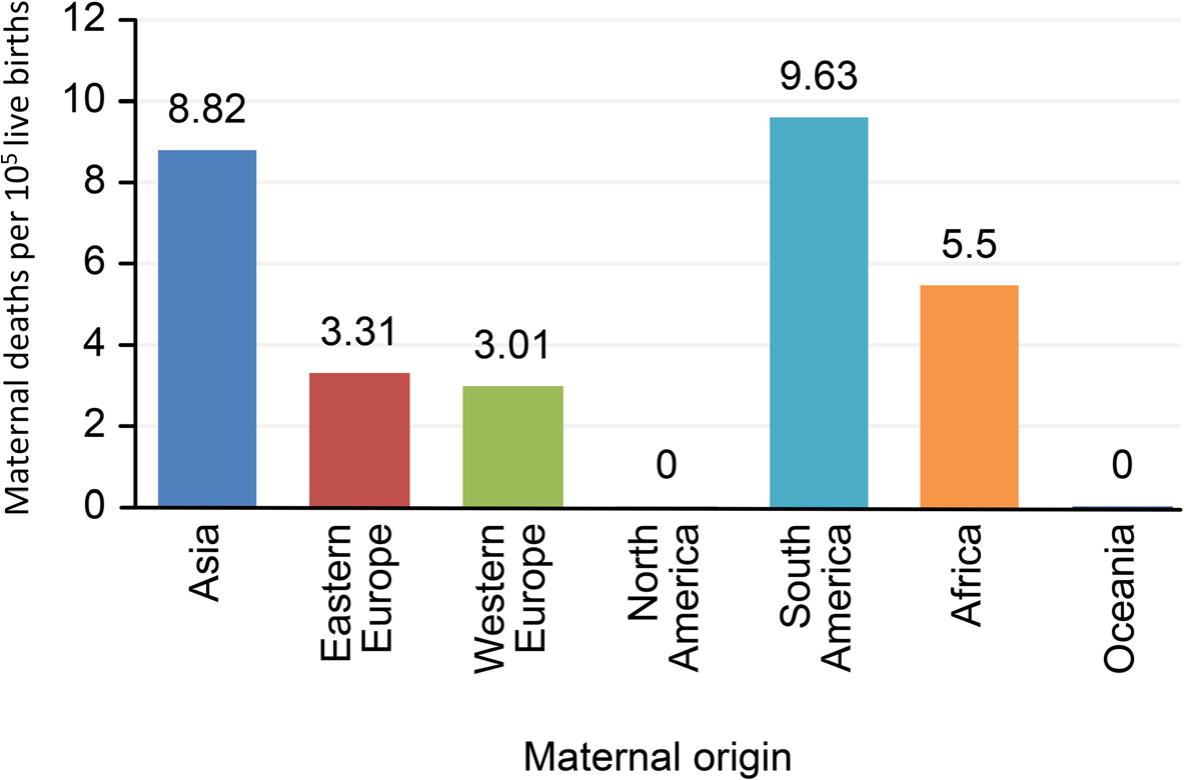
Number of maternal deaths per 10^5^ live births in each group during the studied period

We found statistically significant differences in the proportion of maternal deaths by continent of maternal origin (*p* < 0.01) (Fig. 2). The odds ratio for maternal death in women of non-Spanish origin was 2.19 (95% CI 1.68–2.85) compared to Spanish patients in the univariate analysis.

There were a total of 272 maternal deaths during the study period, with a maternal mortality rate of 3.57 deaths per 100,000 live births. The main causes of death were haemorrhage in 63 cases (23.16%), complications of hypertensive disorders in 52 cases (19.11%), infection/sepsis in 25 cases (9.19%), and amniotic fluid embolism in 25 cases (9.19%). The remaining causes of maternal death and the descriptive analysis of mortality rate by continent of maternal origin and region of Spain where the birth occurred are shown in Table 1.

**Table 1:** Descriptive analysis of maternal mortality rate by ICD-10 cause, maternal continent of origin and region of delivery, Spain 1999–2015 (*n* = 272 deaths)

Variables		Deaths, n (%)	Total live births (n)	Per 10^5^ live births (95% CI)
ICD-10 Cause				
Ectopic pregnancy	O00	4 (1.4)	7,614,878	0.05 (0.00–0.10)
Pregnancy with abortive outcome (excluding ectopic pregnancy)	O01-O08	14 (5.14)	7,614,878	0.18 (0.08–0.28)
Oedema, proteinuria and hypertensive disorders in pregnancy, childbirth and the puerperium	O10-O16	52 (19.11)	7,614,878	0.68 (0.49–0.86)
Haemorrhage	O20 O44.1 O45 O46 O67 O72	63 (23.16)	7,614,878	0.82 (0.62–1.03)
Infection/sepsis	O75.2 O75.3 O85 O86 O41.1	25 (9.19)	7,614,878	0.32 (0.19–0.45)
Obstetric blood-clot embolism	O22.1 O22.3 O22.5 O22.8 O22.9 O87.0 O87.1 O.87.3 O87.8 O87.9 O88	18 (6.61)	7,614,878	0.23 (0.12–0.34)
Amniotic fluid embolism	O88.1	25 (9.19)	7,614,878	0.32 (0.19–0.45)
Complications of anaesthesia	O29 O74 O89	3 (1.1)	7,614,878	0.03 (0.00–0.08)
Rupture of uterus	O71.0 O71.2	12 (4.41)	7,614,878	0.15 (0.06–0.24)
Other direct causes		23 (8.45)	7,614,878	0.30 (0.17–0.42)
Indirect causes: Diseases of the circulatory system complicating pregnancy, childbirth and the puerperium	099.4	7 (2.57)	7,614,878	0.09 (0.02–0.16)
Diseases of the circulatory system complicating pregnancy, childbirth and the puerperium	O98, O99.1–3, 5–9	21 (7.72)	7,614,878	0.27 (0.15–0.39)
Obstetric death of unspecified cause	O95	5 (1.83)	7,614,878	0.06 (0.00–0.12)
Maternal continent of origin				
	Asia	8 (2.9)	90,658	8.82 (2.7–14.93)
	Eastern Europe	7 (2.57)	211,130	3.31 (0.85–5.77)
	Western Europe	198 (72.79)	6,536,936	3.02 (2.6–3.45)
	North America	0	6786	–
	South America	39 (14.33)	404,640	9.63 (6.61–12.66)
	Africa	20 (7.35)	363,556	5.5 (3.09–7.91)
	Oceania	0	600	–
Region				
	Andalucia	87 (31.9)	1,486,731	5.85 (4.62–7.08)
	Aragon	13 (4.77)	198,141	6.56 (2.99–10.12)
	Islas Baleares	8 (2.94)	185,227	4.31 (1.32–7.31)
	Cataluña	42 (15.4)	1,283,792	3.27 (2.28–4.26)
	Canarias	13 (4.77)	316,293	4.11 (1.87–6.34)
	Cantabria	3 (1.1)	84,217	3.56 (0.4–7.59)
	Castilla La Mancha	9 (3.3)	32,8770	2.73 (0.94–4.52)
	Castilla y Leon	12 (4.41)	321,674	3.73 (1.61–5.84)
	Comunidad de Madrid	25 (9.1)	1,148,522	2.17 (1.32–3.02)
	Comunidad Foral de Navarra	1 (0.3)	106,000	0.94 (0.09–2.79)
	Comunidad Valenciana	23 (8.45)	809,828	2.84 (1.67–4.0)
	Extremadura	5 (1.8)	167,177	2.99 (0.36–5.61)
	Galicia	5 (1.8)	351,218	1.42 (0.17–2.67)
	Pais Vasco	3 (1.1)	331,237	0.90 (0.01–1.93)
	Principado de Asturias	9 (3.3)	123,276	7.3 (2.53–12.07)
	Region de Murcia	8 (2.94)	283,099	2.82 (0.86–4.78)
	La Rioja	2 (0.7)	49,780	4.01 (0.01–9.58)
	Ceuta	2 (0.7)	18,481	10.82 (0.4–25.8)
	Melilla	2 (0.7)	21,143	9.45 (0.36–22.5)

Source: Spanish National Institute of Statistics (INE)

Table 2 shows the results of the univariate and multivariate analyses adjusted for the variables described in the study methodology. The region of Spain with the lowest maternal mortality was the Basque Country. Ceuta had the highest mortality, with an OR of 12.11 (95% CI 2.02–72.68). Women whose continent of origin was South America had the highest excess risk, with an OR of 3.92 (95% CI 2.75–5.58). Comparisons of mortality by continent of maternal origin were made with respect to the Western European group, which includes Spain. There were no deaths among patients from North America and Oceania, although the number of women from these countries who gave birth in Spain was very small compared to other groups.

**Table 2:** Maternal mortality risk by region, continent of origin and year of delivery

	Univariate			Multivariate		
	p	OR	95% CI	p	OR	95% CI
Region	< 0.001			< 0.001		
Andalucia	0.001	6.46	2.04–20.42	0.001	6.82	2.16–21.58
Aragon	0.002	7.24	2.06–25.42	0.002	6.96	1.98–24.45
Baleares	0.021	4.77	1.27–17.98	0.034	4.20	1.11–15.86
Cataluña	0.032	3.61	1.12–11.65	0.053	3.18	0.98–10.27
Canarias	0.018	4.54	1.29–15.93	0.022	4.32	1.23–15.16
Cantabria	0.094	3.93	0.79–19.49	0.089	4.01	0.81–19.88
Castilla-La Mancha	0.097	3.02	0.82–11.17	0.098	3.02	0.82–11.15
Catilla-León	0.028	4.12	1.16–14.60	0.026	4.20	1.19–14.90
Com. Madrid	0.151	2.40	0.73–7.96	0.239	2.05	0.62–6.81
Navara	0.972	1.04	0.11–10.01	0.988	0.98	0.10–9.45
Com. Valenciana	0.063	3.14	0.94–10.44	0.075	2.99	0.90–9.95
Extremaduara	0.102	3.30	0.79–13.82	0.080	3.60	0.86–15.06
Galicia	0.536	1.57	0.38–6.58	0.487	1.66	0.40–6.95
País vasco^a^		1.00			1.00	
Asturias	0.002	8.06	2.18–29.78	0.001	8.37	2.27–30.92
Murcia	0.093	3.12	0.83–11.76	0.138	2.73	0.72–10.31
La Rioja	0.103	4.44	0.74–26.55	0.131	3.97	0.66–23.80
Ceuta	0.007	11.95	2.00–71.51	0.006	12.11	2.02–72.68
Melilla	0.010	10.44	1.75–62.51	0.016	9.12	1.50–55.33
Nationality						
Non-Spanish	< 0.001	2.19	1.68–2.85			
Spanish^a^		1.00				
Continent of origin	< 0.001			< 0.001		
Asia	0.003	2.91	1.44–5.91	< 0.001	3.57	1.75–7.30
Eastern Europe	0.814	1.10	0.52–2.33	0.560	1.25	0.59–2.68
Western Europe^a^		1.00		0		
North America	0.982	0.00	–	0.982	0.00	–
South America	< 0.001	3.18	2.56–4.49	< 0.001	3.92	2.75–5.58
Africa	0.011	1.82	1.15–2.88	0.005	1.96	1.22–3.15
Oceania	0.995	0.00	–	0.995	0.00	–
Year	0.698			0.604		
1999	0.191	1.75	0.76–4.04	0.113	1.97	0.85–4.57
2000	0.229	1.67	0.72–3.86	0.146	1.86	0.80–4.31
2001	0.096	1.99	0.89–4.46	0.064	2.15	0.96–4.83
2002	0.280	1.59	0.69–3.67	0.233	1.67	0.72–3.85
2003	0.057	2.15	0.98–4.72	0.048	2.22	1.01–4.87
2004	0.049	2.19	1.01–4.79	0.046	2.22	1.02–4.85
2005	0.138	1.83	0.82–4.08	0.140	1.83	0.82–4.07
2006	0.454	1.38	0.60–3.18	0.478	1.35	0.59–3.13
2007	0.603	1.25	0.54–2.93	0.674	1.20	0.51–2.81
2008	0.045	2.19	1.02–4.71	0.063	2.07	0.96–4.45
2009	0.236	1.63	0.73–3.66	0.290	1.55	0.69–3.47
2010	0.096	1.95	0.89–4.28	0.119	1.87	0.85–4.11
2011	0.423	1.41	0.61–3.25	0.466	1.37	0.59–3.16
2012	0.925	1.04	0.42–2.57	0.969	1.02	0.41–2.50
2013	0.088	2.01	0.90–4.47	0.095	1.98	0.89–4.40
2014^a^		1.00			1.00	
2015	0.210	1.70	0.74–3.88	0.205	1.71	0.75–3.90

^a^Reference

The year 2014 was the one with the lowest risk of maternal death. The highest rates were observed in 2003 (OR 2.22, 95% CI 1.01–4.87) and 2004 (OR 2.22, 95% CI 1.02–4.85).

## Discussion

### Causes of maternal death in Spain

In this study, we found that the main causes of maternal death in Spain during the study period were haemorrhage, complications of hypertensive disorders, infection/sepsis and amniotic fluid embolism.

Several cross-sectional studies have evaluated the main causes of maternal mortality in high income countries such as ours, as well as in impoverished countries. The most relevant study published to date has been the 2015 Global Burden of Disease (GBD) Study, in which a global and regional review of data from 186 countries during the period of 1990–2015 identified the eight main causes of maternal death. This analysis showed that only ten countries achieved the millennium development goal of reducing maternal mortality and identified haemorrhage as the main cause of death [24]. Other relevant causes of maternal death identified in this study were maternal sepsis, hypertensive disorders of pregnancy, obstructed labour and uterine rupture. Global reductions in maternal mortality have not yet reached the initially proposed value of 75%; the GBD study reports reductions of close to 30%. In 2015, more than 250,000 women died during pregnancy or immediately after delivery, most of them from preventable causes.

During the 2011–2013 period in the United States, the maternal mortality rate was 17 deaths per 100,000 live births, higher than the reported rate in Spain during 1999–2015. Data from the United States show an increase in risk with increasing maternal age and differences according to ethnic origin, with a 3.4-fold increase in risk among non-Hispanic black women. In this study, the main causes of maternal death were cardiovascular disease (15.5%), obstetric haemorrhage (11.4%), pulmonary thromboembolism (9.2%), amniotic fluid embolism (5.5%), hypertensive disorders of pregnancy (7.4%) and maternal infection (12.7%) [25]. In the United Kingdom the main cause of maternal death identified in 2012–2014 was cardiovascular disease, with a specific rate of 2 deaths per 100,000 live births [26].

Our data reveal that cardiovascular disease was among the main causes of maternal death in our country (although not the most important), with 46 cases (17% of total maternal deaths). Cardiovascular deaths included 18 cases of thromboembolism, 7 cases classified as indirect causes of circulatory diseases and 21 cases of specific circulatory causes.

Maternal deaths are generally underestimated in countries of the European Union and in the USA, with up to 40–60% inaccurate records of the causes of death, which may explain differences in the reported causes of death in published series [27, 28].

### Maternal mortality in Spain and differences by region

Our results show a very low maternal mortality rate compared with countries similar to ours, such as Norway, which has one of the lowest rates in Europe with 7.2 deaths per 100,000 live births [29].

Even so, we found very significant differences between regions of Spain, with the Basque Country having the lowest mortality rate and Ceuta having the highest risk of death after adjusting for year and maternal origin. These data differ from those previously published by Luque Fernandez MA et al. [23], who showed that Andalucia had the highest risk of maternal death adjusted for origin and maternal age. Our study did not take maternal age into account as an adjustment variable, but our study period was longer by 9 years.

There are notable differences in the public health infrastructure of each region in our country in terms of economic expenditure and level of provision of health services, and these have been exacerbated since the 2008 crisis. The crisis may have diminished the quality of care in the most disadvantaged areas of Spain and jeopardized equity between territories. Similar patterns have been observed in other territories of the European Union [30]. Thus, all countries should increase their awareness of the problem and establish national and local initiatives to reduce inequalities between citizens.

### The excess risk of maternal death in immigrants

The data in our study show an excess risk of maternal death among immigrant women in our country. These results are consistent with findings from the existing literature, showing significant differences in maternal morbidity and mortality according to racial/ethnic origin. In the USA, these differences can be extreme [19].

Possible factors that may contribute to the increased risk of maternal death in certain populations include a higher prevalence of cardiovascular diseases and obesity as well as poorer management of maternal health [31]. Differences in the proportion of perineal lacerations [32], use of tobacco and alcohol [33], gestational diabetes [34], type of anaesthesia used during caesarean [35] and ectopic pregnancy [36] have also been observed when comparing groups by maternal origin.

Some authors have identified hospital factors that may be involved in the unequal distribution of maternal death by ethnic groups. Howell et al. studied morbidity in New York City and found poorer perinatal outcomes among non-Hispanic black women compared with non-Hispanic white patients. This analysis concluded that the poorer obstetric outcomes could have been avoided to some extent in a healthcare centre that typically serves white women during labour [37]. In this regard, disparities at the institutional level should be documented and addressed in such a way that factors that worsen equitable care among pregnant women and that are potentially avoidable can be identified.

Pedersen GS et al. examined whether an increased risk of maternal mortality exists among migrants in Western Europe including 13 studies with more than 42 million women and 4995 maternal deaths. This systematic review showed that immigrant women in Western European countries have a doubled risk of maternal death when compared with indigenous born women [38].

### Strengths and limitations of the study

This study has several strengths. First, it is the most recent analysis of maternal death in our country with data provided by each region and has the longest period of observation among such studies. Additionally, we were able to adjust for relevant sociodemographic variables when quantifying the risk of maternal death in our country and identified the most important causes of maternal death.

Our analysis also has several limitations. We did not have data for very important adjustment variables such as maternal age or the presence of obesity and/or maternal cardiovascular factors that increase the risk of maternal death. We also did not have sociodemographic adjustment variables that would provide a better profile of each pregnant woman, such as the level of fluency in Spanish, duration of living in our country, educational level, degree of social integration, degree of deprivation, access to financial risk protection/health insurance scheme or family structure. This information is generally not recorded nationally or it is variably recorded across regions in our country.

Another important limitation was that we did not include deaths from any obstetric causes occurring after 42 days but less than one year after delivery and deaths from sequelae of obstetric causes occurring one year or more after delivery as recommended in the monitoring of the Sustainable Development Goals (SDGs).

In addition, we did not show data on the level of health service provision in each region or the per capita health investment made by each region. This information could help to better identify possible problems at the institutional level in hospitals and thus help understand differences observed within the same country.

## Conclusions

This study demonstrates an excess risk of maternal death in certain regions and among immigrant women in Spain. The differences observed could be due to disparities in the quality of care in the prenatal period and during childbirth and postpartum care, as well as socio-economic factors related to maternal origin.

Bearing in mind that a significant portion of maternal deaths are potentially preventable, we should implement epidemiological systems in our country to monitor and analyse social and demographic factors that have an important impact on the perinatal prognosis of pregnant women. It would also be important to conduct periodic national analyses of maternal deaths to better understand relevant causes and modifiable risk factors and reduce their incidence.

## Abbreviations

EPMM: Ending preventable maternal mortality
GBD: Global Burden of Disease
HDI: Human development index
INE: Instituto Nacional de Estadistica
SDG: Sustainable Development Goal
USA: United States of America
WHO: World Health Organization
